# Amino-Functionalized Titanate Nanotubes: pH and Kinetic Study of a Promising Adsorbent for Acid Dye in Aqueous Solution

**DOI:** 10.3390/ma15186393

**Published:** 2022-09-15

**Authors:** Débora A. Sales, Paloma N. S. Lima, Lucinaldo S. Silva, Thalles M. F. Marques, Suziete B. S. Gusmão, Odair P. Ferreira, Anupama Ghosh, Yuset Guerra, Alan Í. S. Morais, Roosevelt D. S. Bezerra, Edson C. Silva-Filho, Bartolomeu C. Viana

**Affiliations:** 1 Interdisciplinary Laboratory for Advanced Materials (LIMAV), Materials Science & Engineering Graduate Program, Federal University of Piauí (UFPI), Teresina 64049-550, PI, Brazil; 2 Federal Institute of Maranhão (IFMA), Codó 65400-000, MA, Brazil; 3 Federal Institute of Piauí (IFPI), São João do Piauí 64760-000, PI, Brazil; 4 Laboratório de Materiais Funcionais Avançados (LaMFA), Departament of Physics, Federal University of Ceará (UFC), Fortaleza 60455-900, CE, Brazil; 5 Central Analítica, Federal University of Ceará (UFC), Fortaleza 60455-900, CE, Brazil; 6 Department of Physics, Federal University of Piauí (UFPI), Teresina 64049-550, PI, Brazil; 7 Federal Institute of Education, Science and Technology of Piauí (IFPI), Teresina 64000-040, PI, Brazil

**Keywords:** titanate nanotubes, aminosilane, adsorption, remazol blue R

## Abstract

This work reports the functionalization of sodium titanate nanotubes with amine groups obtained from the reaction of titanate nanotubes with [3-(2-Aminoethylamino)propyl]trimethoxysilane, NaTiNT−2NH, and 3-[2-(2-Aminoethylamino)ethylamino]propyltrimethoxysilane, NaTiNT−3NH. It was verified that the crystalline and morphological structures of NaTiNT were preserved after the functionalization, spectroscopies showed that aminosilane interacted covalently with the surface of NaTiNT, and the incorporation of the aminosilane groups on the surface of NaTiNT can be confirmed. The adsorbent matrices NaTiNT−2NH and NaTiNT−3NH were used to remove the anionic dye from remazol blue R (RB) in aqueous medium, and the highest adsorption capacity was around 365.84 mg g${}^{-1}$ (NaTiNT−2NH) and 440.70 mg g${}^{-1}$ (NaTiNT−3NH) in the range of pH 5.0 to 10.0 and the equilibrium time was reached in 210 min (NaTiNT−2NH) and 270 min (NaTiNT−3NH). Furthermore, the Elovich model, which reports the adsorption in heterogeneous sites and with different activation energies in the chemisorption process, was the most appropriate to describe the adsorption kinetics. Thus, these adsorbent matrices can be used as an alternative potential for dye removal RB in aqueous solution.

## 1. Introduction

The reduction of environmental impacts that derive from industrial development [1,2], in particular water pollution from contaminants such as dyes [3,4,5], metals [6,7,8], and pharmaceuticals [9,10], has been the subject of several studies in recent years. Among the contaminants, basic [11,12] and anionic [13,14,15] dyes can cause several problems to living organisms due to their carcinogenic and mutagenic characteristics, being acid dyes (for example, remazol blue dye (RB) ) with high water solubility and toxicity, making it difficult to remove from wastewater [16]. Furthermore, the pollution resulting from this class of compounds harms several biochemical processes in living beings [1]. In this context, adsorption is a promising method against the challenge of environmental pollution [16,17,18,19]. The adsorption process uses various materials as adsorbents, such as clays, activated carbon, biopolymers, nanomaterials, among others [20,21,22]. Thus, the development of new materials that can be used as adsorbents with high adsorption capacity represents an important action to mitigate the impacts of environmental pollution [23]. In this scenario, titanate nanotubes are very efficient adsorbents for basic (cationic) dyes due to their high surface area and cation exchange behaviors [11,12,13]. However, sodium titanate nanotubes (NaTiNTs) have practically no attraction for acid (anionic) dyes, and their functionalization with amine groups is of fundamental importance to be used as adsorbents for anionic dyes in aqueous media, because adsorption can be favored by electrostatic interactions due these groups being easily protonated under acidic conditions and the acid dye (for example, RB) is negatively charged [15]. Because of this, amino-functionalized titanate nanotubes are versatile adsorbents that have been used to remove acid dyes in aqueous solution [13,14,15], due to the combined action of high surface area functional groups decoration [15,24]. Thus, due to the positive characteristics of amino-functionalized titanate nanotubes in relation to the adsorption of acid dyes from an aqueous medium, more studies are needed regarding the production of new routes of synthesis of functionalized titanate nanotubes with amino groups and their applications in the adsorption of acidic dyes from the environment. Therefore, the production of [3-(2-Aminoethylamino) propyl]trimethoxysilane and 3-[2-(2-Aminoethylamino)ethylamino] propyltrimethoxysilane functionalized titanate nanotubes and their application in the adsorption of acid dyes is necessary, because there are no studies in the literature related to this route of synthesis of this adsorbent for application in the adsorption of acid dyes from the aqueous medium. Thus, this work describes the feasibility of using sodium titanate nanotubes (NaTiNTs) with amine groups (NaTiNT−2NH and NaTiNT−3NH), obtained through the reaction of NaTiNTs with [3-(2-Aminoethylamino)propyl] trimethoxysilane and 3-[2-(2-Aminoethylamino)ethylamino] propyltrimethoxysilane, characterizes them by XRD, BET, SEM, EDS, Raman, FTIR, and XPS, and applies them in the adsorption of the acid dye remazol blue R (RB) with the variation of pH and contact time. In addition, the experimental results of kinetics were fitted to pseudo-first-order, pseudo-second-order and Elovich nonlinear kinetic models.

## 2. Materials and Methods

### 2.1. Materials

This study used the following materials: titanium dioxide (TiO${}_{2}$, Sigma-Aldrich, São Paulo, Brazil, 99.8%), sodium hydroxide (NaOH, Dinâmica, Indaiatuba, Brazil, 98%), [3-(2-Aminoethylamino)propyl] trimethoxysilane, 3-[2-(2-Aminoethylamino) ethylamino] propyltrimethoxysilane (Sigma-Aldrich, São Paulo, Brazil, 97%), dichloromethane (CCl${}_{2}$H${}_{2}$, Sigma-Aldrich, São Paulo, Brazil, 97%), hydrochloric acid (HCl, Dinâmica, Indaiatuba, Brazil, 36.5–38%), potassium nitrate (KNO${}_{3}$, Química Moderna, Barueri, Brazil, 99%), remazol blue dye R (RB, Vetec, Duque de Caxias, Brazil), and deionized water. All reagents were analytical grade and no previous purification was need.

### 2.2. Synthesis of Sodium Titanate Nanotubes

For the production of sodium titanate nanotubes (NaTiNT), 3.00 g of TiO${}_{2}$ (anatase) were placed in 90 mL of 10.0 mol L${}^{-1}$ NaOH. Subsequently, this solution was placed in a Teflon reactor for 96 h at 140 °C. Finally, after cooling the system, a white solid was formed, which was washed several times with deionized water until the pH was between 10.0 and 12.0, and, subsequently, this material was dried in vacuo for 24 h [15].

### 2.3. Synthesis of Modified Nanotubes

The modified nanotubes (NaTiNT−2NH and NaTiNT−3NH) were produced using the method described in the literature by Ref. [6]. A mixture composed of NaTiNT (0.25 g), CCl${}_{2}$H${}_{2}$ (50.0 mL) and aminosilane (0.50 mL) [3-(2-Aminoethylamino) propyl] trimethoxy-silane (TMSDA) (NaTiNT−2NH) or 3-[2-(2-Aminoethylamino) ethylamino] propyltrimethoxy-silane (TMSTA) (NaTiNT−3NH) was suspended under stirring for 3 h at room temperature [6].

### 2.4. Point of Zero Charge ($pH_{pzc}$)

The point of zero charge ($pH_{pzc}$) was carried out using the method described in the literature [25,26]. Twenty mg of the solid (NaTiNT, NaTiNT−2NH or NaTiNT−3NH) were placed in Erlenmeyers containing 20 mL of NaCl solution (0.1 mol L${}^{-1}$) with different pHs (1.0 to 12.0). These pHs were regulated with the addition of HCl (0.1 mol L${}^{-1}$) and/or NaOH (0.1 mol L${}^{-1}$) solutions. Soon after, the Erlenmeyers were left for 24 h under agitation (140 rpm). Subsequently, the suspensions were centrifuged (3500 rpm/5 min), and the final pH ($pH_{f}$) was measured. e ΔpH versus $pH_{i}$ graph was plotted, where ΔpH is the difference between the initial and final pH ($\Delta pH=pH_{i}-pH_{f}$).

### 2.5. Remazol Blue R Adsorption

The RB dye adsorption assays with the adsorbents NaTiNT−2NH and NaTiNT−3NH were carried out using 20 mg of the modified nanotubes with 20 mL of the dye solution (room temperature). The dye concentration (initial and final) was determined by UV-Vis, as shown in the literature [27,28]. The adsorption process varying the pH occurred with: the dye concentration—1000 mg L${}^{-1}$, the time—24 h, the temperature—25 °C, and the pH—1.0 to 12.0. The adsorption process varying the time occurred with: concentration—1000 mg L${}^{-1}$, time—0 to 360 min, temperature—25 °C and pH—6.5 (natural pH of the dye solution). The equilibrium adsorption amount ($Q_{e}$) of RB was calculated according to Equation (1):

(1) $$Q_{e}=\frac{C-C_{e}}{M}V$$

where $Q_{e}$ is the equilibrium adsorption amount (mg g${}^{-1}$), $C_{0}$ and $C_{e}$ are the initial and equilibrium concentrations of remazol blue (mg L${}^{-1}$), respectively, *M* is the mass of adsorbents (g), and *V* is the volume of dye solution (L).

### 2.6. Kinetic Models

Equation (2) shows the nonlinear Equation used to fit the experimental data to the pseudo-first order model (Lagergren):

(2) $$q_{t}=q_{e,cal}\left[1-\exp\left(-k_{1}t\right)\right]$$

where $q_{e,cal}$ (mg g${}^{-1}$) is the adsorbed amount per gram of adsorbent, $q_{t}$ (mg g${}^{-1}$) is the adsorbed amount per gram of adsorbent at time *t* (mim), and $k_{1}$ (min${}^{-1}$) is the pseudo-first-order velocity constant [29]. Equation (3) presents the non-linear Equation used to perform the experimental adjustments to the pseudo-second order model ([30]):

(3) $$q_{t}=\frac{k_{2}q_{e,cal}^{2}t}{1+k_{2}q_{e,cal}t}$$

where $q_{t}$ (mg g${}^{-1}$) is the adsorbed amount per gram of adsorbent at time *t* (mim) and $k_{2}$ (g mg${}^{-1}$ min${}^{-1}$) is the pseudo-second-order rate constant [30]. The Elovich model is represented by the following equation in nonlinear form (Equation (4)):

(4) $$q_{t}=\frac{1}{\beta }\ln\left(\alpha \beta t\right)$$

where qt (mg g${}^{-1}$) is the adsorbed amount per gram of adsorbent at time *t* (mim), β (g mg${}^{-1}$) is the adsorption constant and α (mg g${}^{-1}$min${}^{-1}$) is the initial adsorption rate constant [31].

### 2.7. Characterizations

X-ray diffraction (XRD) patterns were obtained with a Bruker D8 diffractometer, using CuKα radiation (λ=1.5406 Å) operating at 30 mA and 40 kV. A scan rate of 2° min${}^{-1}$ was used and the 2° range was 5–70 degrees. Surface area measurements were performed using a BELSORP—mini II, BEL JAPAN equipment. Initially, samples were treated at 105 °C for 14 h under N2 flow. The nitrogen adsorption/desorption (BET) study was carried out at 77 K with a maximum pressure of 1 atm and a relative pressure of 0.99. Raman spectroscopy was performed in a confocal Raman spectrometer, Bruker Senterra, with objective lenses with 50× and 785 nm laser excitation source. Low laser power density was used to prevent the sample from overheating. A spectral resolution of 3 cm${}^{-1}$ was used and the range used was 50–3700 cm${}^{-1}$. Fourier transform infrared (FTIR) spectroscopy was recorded using a KBr configuration on a Vertex 70 spectrometer (Bruker company). A total of 64 scans and a resolution of 4 cm${}^{-1}$ were used to obtain spectra with good signal-to-noise ratios. The range used was 400–4000 cm${}^{-1}$. The morphology of the adsorbent was investigated by scanning electron microscopy (SEM) using a FIB-SEM TESCAN MIRA3 microscope operating at 200 keV, coupled with an energy dispersive X-ray spectrometer (EDS). The samples were prepared by placing an aqueous suspension of the material powder on a grid of the copper coated with carbon and allowing water to evaporate at room temperature. X-ray photoemission (XPS) spectra were obtained with a Scienta Omicron ESCA + spectrometer system equipped with an EA 125 hemispherical analyzer and a monochrome Xm 1000 X-ray source in Al Kα (1486.7 eV). For corrections in peak shifts due to the remaining charge effect, the binding energy of all spectra was scaled using the main peak of C1s at 284.5 eV as reference [15]. Dye concentration was quantified in a UV-VIS spectrophotometer (Cary 60, Agilent Technologies, Santa Clara, CA, USA) at a wavelength (λ) of 593 nm, from a calibration curve.

## 3. Results and Discussion

### 3.1. Material Characterizations

**Figure 1:**
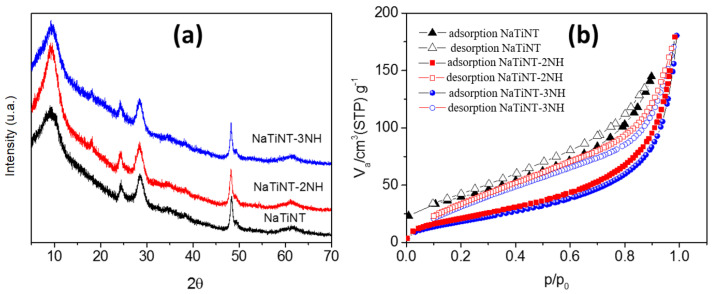
(**a**) X-ray diffraction patterns of sodium titanate nanotubes as synthesized (NaTiNT) and (**b**) of amino-functionalized titanate nanotubes (NaTiNT−2NH and NaTiNT−3NH).

The X-ray diffraction data of the materials (NaTiNT, NaTiNT−2NH, and NaTiNT−3NH) are shown in Figure 1a. The diffractogram profile of the NaTiNT sample highlights four peaks, which can be related to the crystalline planes of Na${}_{2}$Ti${}_{3}$O${}_{7}\cdot$ nH${}_{2}$O indexed as (200), (110), (211), and (020), respectively [32,33]. These peaks are in agreement with data published in the literature [34,35,36,37]. Figure 1 shows the XRD of the nanotubes (NaTiNT, NaTiNT−2NH, and NaTiNT−3NH). From the figure, it can be seen that the crystalline structure of the titanate nanotubes was preserved after the incorporation reaction of the amino groups due to the similarity of the diffractograms of the NaTiNT, NaTiNT−2NH, and NaTiNT−3NH samples [15,38]. XRD shows that there is no excess of aminosilanols in the functionalized nanotubes due to the absence of aminosilanol reflections in the diffractograms [24]. From the nitrogen adsorption-desorption studies of the materials (NaTiNT, NaTiNT−2NH, and NaTiNT−3NH), shown in Figure 1b, it can be seen that the isotherms are of type IV with hysteresis of type H3, which indicates the presence of mesopores [15]. The BET surface area and total pore volume of NaTiNT−2NH and NaTiNT−3NH were lower than that of NaTiNT (Table 1), which indicates that the silane molecules efficiently coat the surface of the adsorbents, thus promoting a reduction in available surface area and pore volume. Some similar results are also shown in the literature [6].

**Figure 2:**
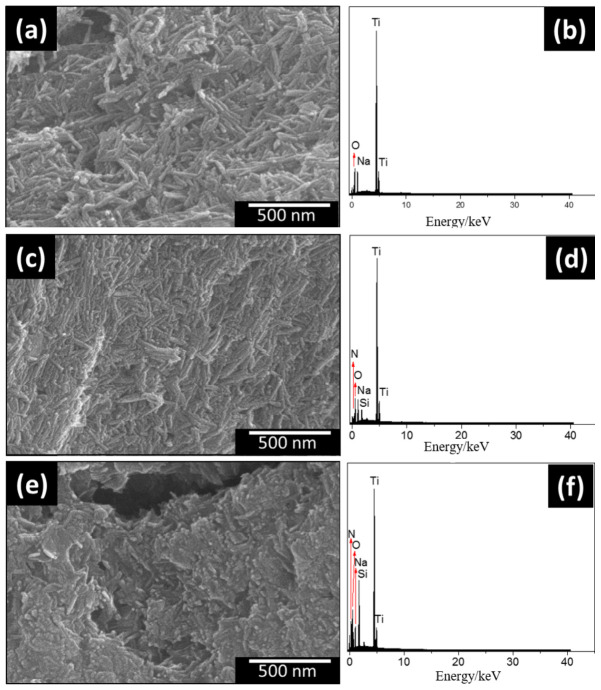
Scanning electron microscopy images and dispersion analysis by X-ray spectroscopy of sodium titanate nanotubes as synthesized. (**a**,**b**) NaTiNT, (**c**,**d**) amino-functionalized titanate nanotubes (NaTiNT−2NH), (**e**,**f**) NaTiNT−3NH.

The micrographs shown in Figure 2 illustrate the morphological characterization of titanate nanotubes as synthesized and after chemical modification. In common, all products had predominantly agglomerated materials and a compact and very porous fibrous structure, with fibers randomly intertwined or arranged in parallel bundles. As illustrated in Figure 2a,c,e, it was not possible to make a morphological distinction between the samples. The chemical compositions present in the samples were investigated by dispersion analysis by X-ray spectroscopy (EDS). For NaTiNT, signs of Ti, O, and Na were observed [37]. The EDS spectra of the nanotubes after modification (Figure 2d,f) showed additional signals corresponding to Si and N when compared to NaTiNT (Figure 2b), indicating the presence of aminosilanols in the samples—NaTiNT−2NH and NaTiNT−3NH.

**Figure 3:**
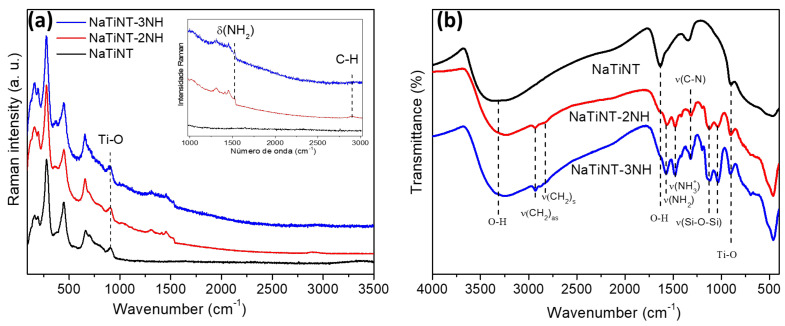
(**a**) Raman spectra and FTIR spectra (**b**) of NaTiNT, NaTiNT−2NH, and NaTiNT−3NH.

The Raman spectra, shown in Figure 3a, exhibit vibrational characteristics typical of NaTiNT. From this Figure, it can be seen that the functionalization did not change the characteristic vibrational modes of the titanate nanotubes due to the preservation of the vibrational modes of NaTiNT in the spectra of NaTiNT−2NH and NaTiNT−3NH [39]. Furthermore, the spectra of the functionalized samples show bands (1000–3500 cm${}^{-1}$) with characteristic vibrations of organic groups, indicating that there are vibrational modes of the aminosilanol groups present on the surface of the titanate nanotubes [15]. Figure 3b shows the FTIR spectra of the materials (NaTiNT, NaTiNT−2NH, and NaTiNT−3NH). All samples have a broad band in the range 2600 to 3700 cm${}^{-1}$, related to stretching vibrations of the OH groups present in the nanotubes and a strong band at 1637 cm${}^{-1}$, which can be related to the flexural H−O−H mode of vibration of water molecules. In addition, a band at approximately 900 cm${}^{-1}$ can be observed, which is attributed to Ti−O bonds (nonbridging oxygen bonds) [38,40]. After the functionalization reaction of NaTiNT with aminosilanol molecules, significant changes can be seen that show the proposed functionalization to obtain NaTiNT−2NH and NaTiNT−2NH. The functionalized nanotubes showed a band at 1478 cm${}^{-1}$, referring to the symmetric bending deformation of $NH_{3}^{+}$ [39]. Another band at 1570 cm${}^{-1}$ was related to $NH_{2}$ deformation vibration. This confirms the presence of the amino-terminal groups of the aminosilane molecules [6]. The N−H stretch vibration (band at 3500 cm${}^{-1}$) cannot be observed due to the superposition of the O-H stretch band. The $CH_{2}$ vibrations of the aminopropyl segment can be observed in the range of 1450–1300 cm${}^{-1}$ [37]. The bands at 1122 cm${}^{-1}$ and 1036 cm${}^{-1}$ are related to the vibrations of the Si-O-Si groups [38,39]. These results prove the incorporation of aminosilane groups on the surface of titanate nanotubes of TiNT−2NH and TiNT−3NH materials [15].

**Figure 4:**
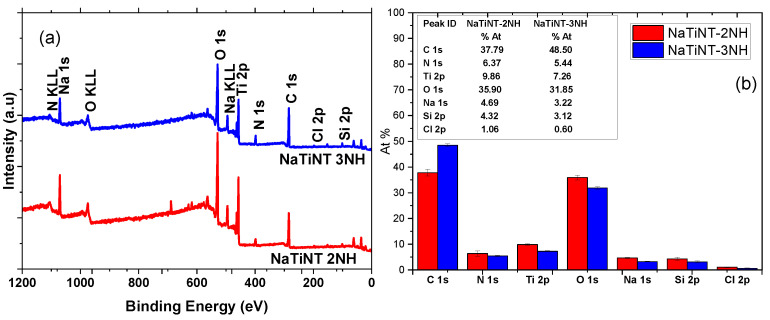
(**a**) Full scan X-ray photoelectron spectroscopy of the samples (NaTiNT−2NH and NaTiNT−3NH) and (**b**) XPS elemental quantification results of the samples.

The results of the X-ray photoelectron spectroscopy (XPS) of the materials (NaTiNT− 2NH and NaTiNT−3NH) are shown in Figure 4. From XPS, the presence of the peaks attributed to the electrons Ti, O, and Na, referring to the starting material (NaTiNT), can be observed. In addition, the presence of the peaks attributed to the N and Si electrons can be observed. The presence of these peaks confirms the incorporation of aminosilane groups on the surface of the nanotubes after the modification reaction in the materials (NaTiNT−2NH and NaTiNT−3NH) [15]. The peak related to the Cl electrons, present in the spectra of materials, is related to a small amount of $CCl_{2}H_{2}$ residue used as a solvent in the modification reaction.

**Figure 5:**
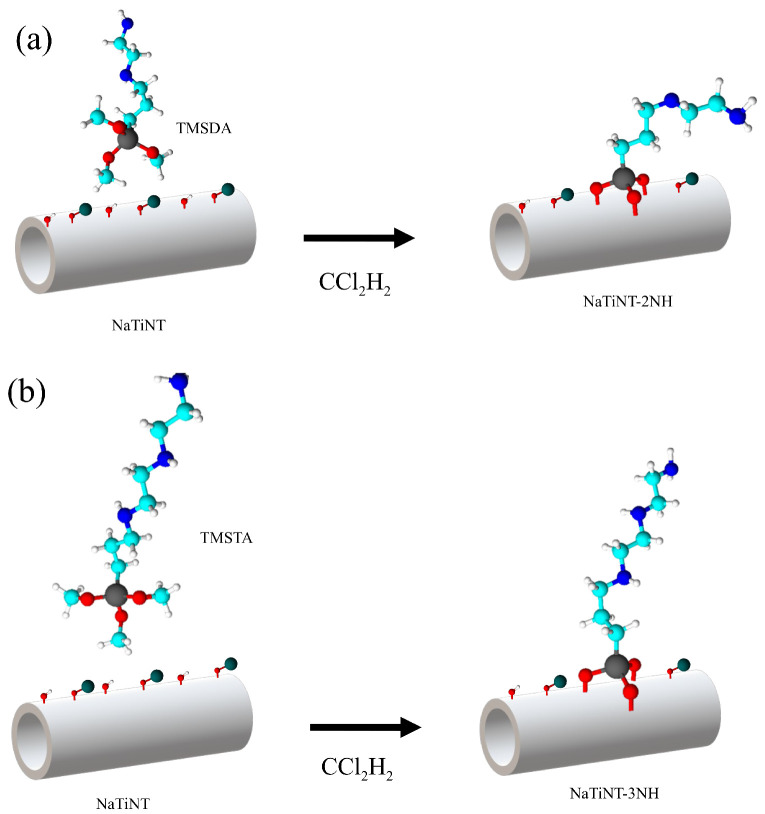
Proposed reaction scheme for the synthesis of (**a**) NaTiNT−2NH and (**b**) NaTiNT−3NH from NaTiNT.

Thus, the characterization results indicated that the grafting reactions of aminosilane groups on the surface of NaTiNT was performed by the condensation of silanol units and hydroxyl units present on the surface of the nanotubes, as shown in Figure 5.

**Figure 6:**
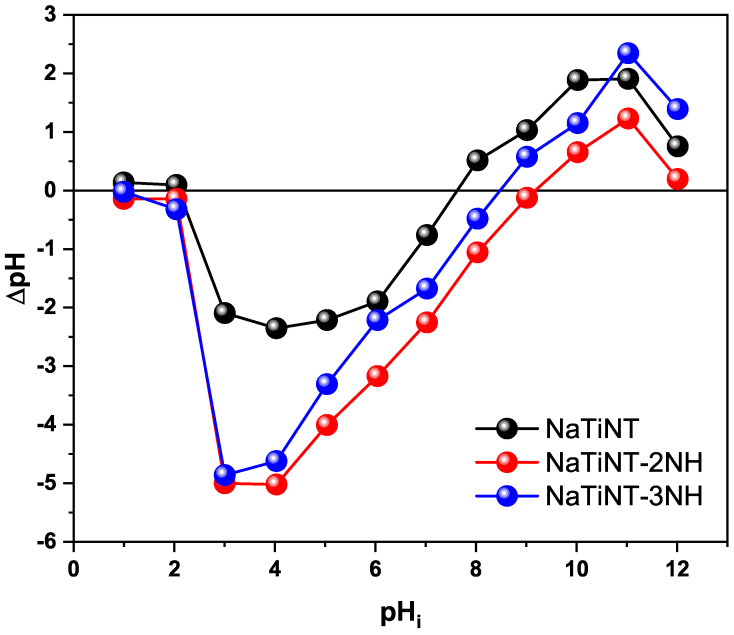
$pH_{pcz}$ of the NaTiNT, NaTiNT−2NH, and NaTiNT−3NH matrix.

Figure 6 shows the curve of the point of zero charge, pHpzc, of the NaTiNT, NaTiNT−2NH, and NaTiNT−3NH matrices, which shows that the main difference is in the $pH_{pzc}$: 7.6 (NaTiNT), 9.2 (NaTiNT−2NH), and 8.4 (NaTiNT−3NH). This result shows that the alteration in the $pH_{pzc}$ of the modified materials is related to the incorporation of aminosilanes groups on the surface of NaTiNT that promote the increase of the positive charge density due to the protonation of the nitrogen present in these groups [15,41]. In general, at $pH<pH_{pcz}$, the adsorbent matrix can be used in the anionic dye adsorption process (for example, RB dye, because it is negatively charged). At $pH>pH_{pcz}$, the matrix can be used in the adsorption of cationic dye (for example, Methylene Blue dye), as it is a negatively charged surface [42].

### 3.2. Adsorption Studies

**Figure 7:**
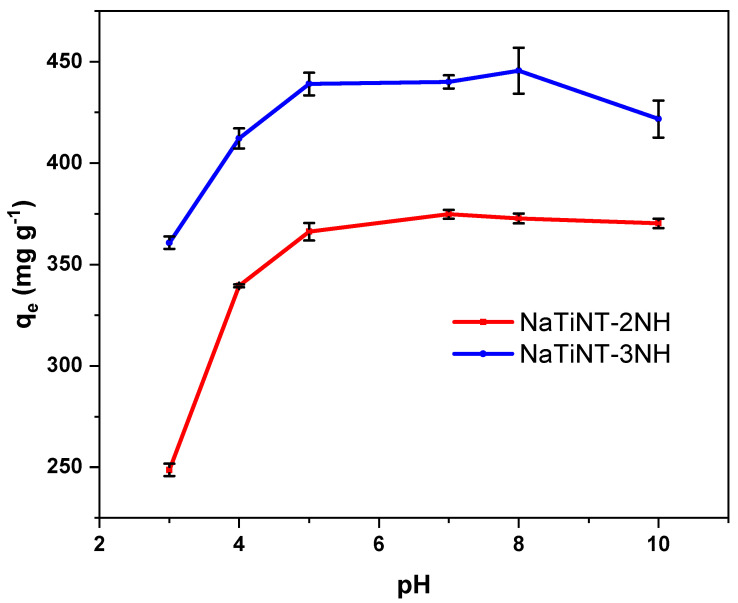
The effect of pH on RB adsorption using the adsorbent NaTiNT−2NH and NaTiNT−3NH.

**Figure 8:**
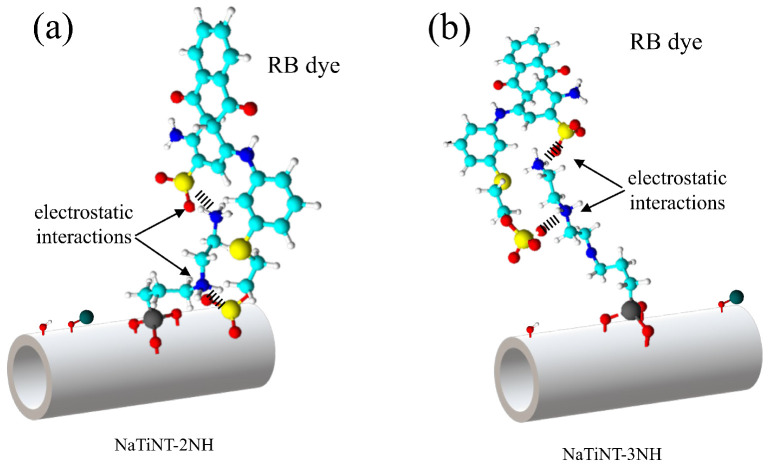
Scheme of the adsorption process of RB on (**a**) NaTiNT−2NH and (**b**) NaTiNT−3NH.

The study of pH in the RB dye adsorption process using adsorbent matrices (NaTiNT−2NH and NaTiNT−3NH) is shown in Figure 7. From the graphs, it can be seen that as the pH increases, there is an increase in the adsorption of the RB dye by the two materials until pH 5, and the adsorption capacity was around 365.84 mg g${}^{-1}$ for NaTiNT−2NH and 440.70 mg g${}^{-1}$ for NaTiNT−3NH. From pH 5 onwards, the amount of dye adsorbed by the two materials remains practically constant until pH 10. This result is due to the interactions that occur between the adsorbent matrices and the dye. At $pH<pH_{pzc}$, the adsorbent matrices (NaTiNT−2NH and NaTiNT−3NH) retain protons from the medium, and present positive charges on their surface due to the protonation of amine groups. Thus, as the anionic dye RB has a negative charge in an aqueous medium at these pHs, the adsorption process, in the two adsorbent matrices, occurs mainly through electrostatic interactions, as shown in Figure 8. The presence of hydrogen bonds and Van der Waals force during adsorption is also possible, as shown in some works [3,15,43]. The decrease in the amount of dye adsorbed by materials at a pH below 5 is due to the presence of excess H+ ions. The excess of H+ ions prevent the interaction between the adsorbent materials and the RB molecules, because these ions interact electrostatically with the RB molecules (anionic molecules) making interactions with the adsorbent materials impossible [1,18,20]. This result is important because the pH range mentioned above covers the pH that was used to perform the adsorption kinetics tests (pH 6.5).

**Figure 9:**
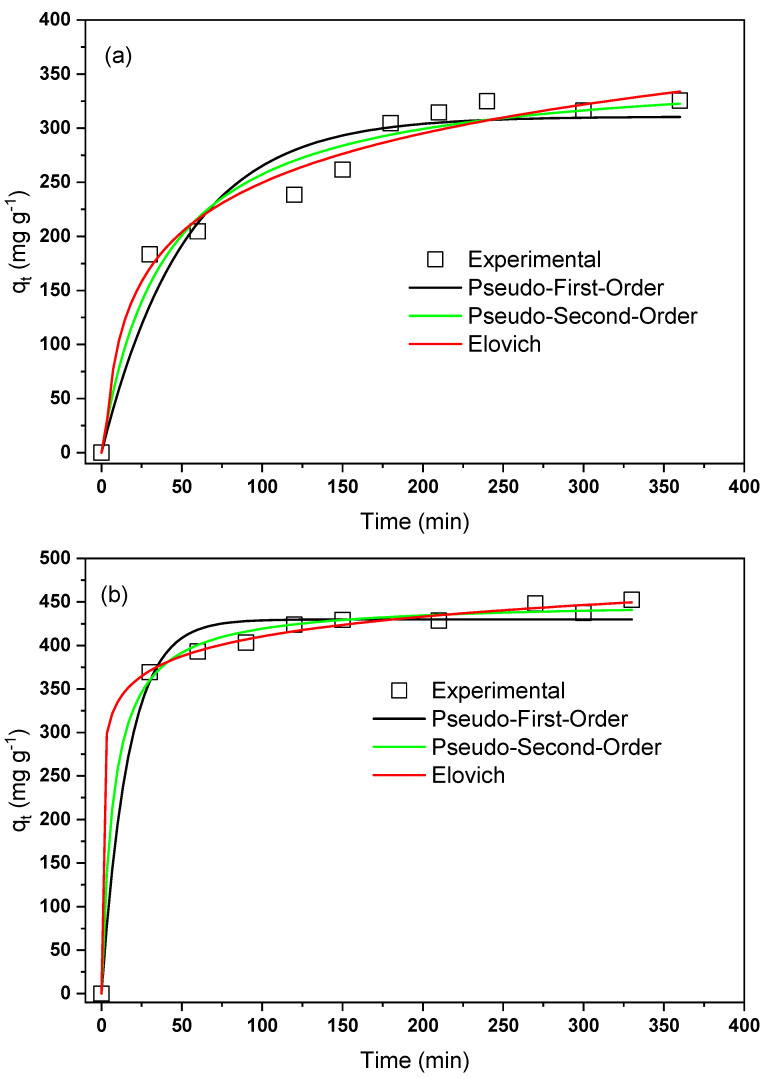
Influence of the contact time on RB dye adsorption using nonlinear fits in kinetic models (**a**) NaTiNT−2NH and (**b**) NaTiNT−3NH.

Figure 9 shows the study of the influence of time on the adsorption of remazol blue dye (RB) using the surface of NaTiNT−2NH and NaTiNT−3NH. It can be observed that for the functionalized nanotubes, after 210 min (NaTiNT−2NH) and 270 min (NaTiNT−3NH) of contact with the RB dye, the amount adsorbed is almost constant, that is, this indicates that this time is sufficient for the adsorption equilibrium to occur. With these results, it is observed that after the proposed modifications in the nanotubes, the equilibrium between adsorbent/adsorbate occurs longer in NaTiNT−3NH, most likely due to the increase in adsorption sites with a greater incorporation of amine groups, which favors the greater capacity of adsorption of the dye in this matrix [15].

Table 2 shows the kinetic parameters obtained for the adsorption of RB dye in NaTiNT−2NH or NaTiNT−3NH adsorbent matrices. From the results obtained, it can be observed that the Elovich model presented the best fit, as it presented the highest value of the correlation coefficient ($R^{2}=0.9757$ (NaTiNT−2NH) and 0.9982 (NaTiNT−3NH)) compared to the other models. Thus, this model, which reports the adsorption in heterogeneous sites and with different activation energies in the chemisorption process, is the most appropriate to describe the kinetic behavior of the adsorption systems discussed, as already observed in the adsorption of the remazol red dye in the surface of pure cellulose [15,42].

The two adsorbent materials of amino-functionalized titanate nanotubes showed promise for the removal of acid dyes, such as acid dye remazol blue R (RB), from aqueous media. The results presented in this work were better than some results in the literature, as shown in Table 3.

## 4. Conclusions

The reactions to obtain the adsorbent matrices of amino-functionalized sodium titanate nanotubes (NaTiNT−2NH and NaTiNT−3NH) were confirmed with the proposed characterizations. Through XRD and SEM, it was verified that the crystal and morphological structures of NaTiNT were preserved after the functionalizations. Raman and FTIR verified that the aminosilane interacted covalently with the surface of NaTiNT. Through XPS the incorporation of the amino silane groups on the NaTiNT surface was confirmed in the materials (NaTiNT−2NH and NaTiNT−3NH). The RB dye adsorption experiments using adsorbents (NaTiNT-2NH and NaTiNT-3NH) in aqueous solution showed that the highest adsorption capacity was around 365.84 mg g-1 (NaTiNT−2NH) and 440.70 mg g${}^{-1}$ (NaTiNT−3NH) in the range of pH 5.0 to 10.0 and the equilibrium time was reached in 210 min (NaTiNT−2NH) and 270 min (NaTiNT−3NH). Furthermore, the Elovich model, which reports the adsorption in heterogeneous sites and with different activation energies in the chemisorption process, was the most appropriate to describe the adsorption kinetics. The adsorbents NaTiNT−2NH and NaTiNT−3NH can be used as alternative materials for the removal of acid dyes (for example, RB) in aqueous solution, since the matrices have good adsorption capacity.

## Figures and Tables

**Table 1:** Nitrogen adsorption-desorption analysis of samples, surface area (BET), diameter and volume of pores.

Samples	$S_{\mathrm{Bet}}$ (m${}^{2}$ g${}^{-1}$)	$D_{\mathrm{av}}$ (nm)	$V_{t}$ (cm${}^{3}$ g${}^{-1}$)
NaTiNT	147.5	10.9	0.401
NaTiNT−2NH	84.2	13.2	0.277
NaTiNT−3NH	74.6	14.9	0.278

*D_av_* represents the average diameter the pores of the adsorbents and *V_t_* represents the pore volume of the adsorbents.

**Table 2:** Kinetic parameters obtained with the Pseudo-First-Order, Pseudo-Second-Order, and Elovich models for RB adsorption on NaTiNT−2NH and NaTiNT−3NH.

Pseudo-First-Order			
**Adsorbent**	$k_{1}$ (min${}^{-1}$)	$q_{e(cal)}$ (mg g${}^{-1}$)	$R^{2}$
NaTiNT−2NH	0.019	310.623	0.9309
NaTiNT−3NH	0.059	429.958	0.9845
**Pseudo-Second-Order**			
**Adsorbent**	$k_{2}$ (g mg${}^{-1}$ min${}^{-1}$)	$q_{e(cal)}$ (mg g${}^{-1}$)	$R^{2}$
NaTiNT−2NH	$7.18\times 10^{-5}$	357.477	0.9635
NaTiNT−3NH	$2.93\times 10^{-4}$	450.960	0.9961
**Elovich**			
**Adsorbent**	*A* (g mg${}^{-1}$ min${}^{-1}$)	β (mg g${}^{-1}$)	$R^{2}$
NaTiNT−2NH	29.20	0.015	0.9757
NaTiNT−3NH	88149.07	0.030	0.9982

**Table 3:** Types of adsorbents used to remove acid dye remazol blue R (RB).

Adsorbent	Maximum Amount Adsorbed (mg g${}^{-1}$)	Reference
Magnetite nanoparticles	74.40	[44]
ZnO nanoparticles	38.02	[45]
Mesoporous activated carbon	33.50	[46]
Chitosan-Glyoxal/Kaolin Clay Composite	284.90	[47]
Magnetic chitosan-Glutaraldehyde/Zinc Oxide/Fe_3_O_4_ Nanocomposite	179.70	[48]
Marine alga “Bifurcaria bifurcata”	88.70	[49]
Mesoporous Silica Nanoparticles	225.0	[50]
Borax cross-linked Jhingan gum hydrogel	9.88	[51]
Magnesium hydroxide coated bentonite	47.21	[52]
Polypyrrole-coated magnetic nanoparticles	112.36	[53]
NaTiNT−2NH	365.84	This work
NaTiNT−3NH	440.70	This work

## References

[1] Dos Santos Silva L., de Oliveira Carvalho J., de Sousa Bezerra R.D., da Silva M., Ferreira F., Osajima J., da Silva Filho E. (2018). Potential of Cellulose Functionalized with Carboxylic Acid as Biosorbent for the Removal of Cationic Dyes in Aqueous Solution. Molecules.

[2] Li C., He Y., Zhou L., Xu T., Hu J., Peng C., Liu H. (2018). Fast adsorption of methylene blue, basic fuchsin, and malachite green by a novel sulfonic-grafted triptycene-based porous organic polymer. RSC Adv..

[3] Silva L.S., Silva M.S., Ferreira F.J.L., Lima L.C.B., Bezerra R.D.S., Citó A.M.G.L., Osajima J.A., Silva Filho E.C. (2018). Effective Removal of the Remazol Yellow GR Dye Using Cellulose Functionalized by Basic Groups. Water Air Soil Pollut..

[4] Chatterjee S., Chatterjee S., Chatterjee B.P., Das A.R., Guha A.K. (2005). Adsorption of a model anionic dye, eosin Y, from aqueous solution by chitosan hydrobeads. J. Colloid Interface Sci..

[5] Subbaiah M.V., Kim D.S. (2016). Adsorption of methyl orange from aqueous solution by aminated pumpkin seed powder: Kinetics, isotherms, and thermodynamic studies. Ecotoxicol. Environ. Saf..

[6] Wang L., Liu W., Wang T., Ni J. (2013). Highly efficient adsorption of Cr(VI) from aqueous solutions by amino-functionalized titanate nanotubes. Chem. Eng. J..

[7] Niu G., Liu W., Wang T., Ni J. (2013). Absorption of Cr(VI) onto amino-modified titanate nanotubes using 2-Bromoethylamine hydrobromide through SN2 reaction. J. Colloid Interface Sci..

[8] Wang H., Zheng L., Liu G., Zhou Y. (2018). Enhanced adsorption of Ag+ on triethanolamine modified titanate nanotubes. Colloids Surfaces A Physicochem. Eng. Asp..

[9] Dobó D.G., Berkesi D., Kukovecz Á. (2017). Morphology conserving aminopropyl functionalization of hollow silica nanospheres in toluene. J. Mol. Struct..

[10] Bergmann C.P., Machado F.M. (2015). Carbon Nanomaterials as Adsorbents for Environmental and Biological Applications.

[11] Sandoval A., Hernández-Ventura C., Klimova T.E. (2017). Titanate nanotubes for removal of methylene blue dye by combined adsorption and photocatalysis. Fuel.

[12] Subramaniam M.N., Goh P.S., Abdullah N., Lau W.J., Ng B.C., Ismail A.F. (2017). Adsorption and photocatalytic degradation of methylene blue using high surface area titanate nanotubes (TNT) synthesized via hydrothermal method. J. Nanopart. Res..

[13] Lee C.K., Liu S.S., Juang L.C., Wang C.C., Lyu M.D., Hung S.H. (2007). Application of titanate nanotubes for dyes adsorptive removal from aqueous solution. J. Hazard. Mater..

[14] Juang L.C., Lee C.K., Wang C.C., Hung S.H., Lyu M.D. (2008). Adsorptive Removal of Acid Red 1 from Aqueous Solution with Surfactant Modified Titanate Nanotubes. Environ. Eng. Sci..

[15] Marques T.M., Sales D.A., Silva L.S., Bezerra R.D., Silva M.S., Osajima J.A., Ferreira O.P., Ghosh A., Silva Filho E.C., Viana B.C. (2020). Amino-functionalized titanate nanotubes for highly efficient removal of anionic dye from aqueous solution. Appl. Surf. Sci..

[16] Elwakeel K.Z., El-Bindary A.A., Ismail A., Morshidy A.M. (2016). Sorptive removal of Remazol Brilliant Blue R from aqueous solution by diethylenetriamine functionalized magnetic macro-reticular hybrid material. RSC Adv..

[17] Sheng G., Linghu W., Chen Z., Xu D., Alsaedi A., Shammakh W., Monaquel S., Sheng J. (2016). Sequestration of selenate and selenite onto titanate nanotube: A combined classical batch and advanced EXAFS approach. Environ. Nanotechnol. Monit. Manag..

[18] Silva L.S., Lima L.C., Silva F.C., Matos J.M.E., Santos M.R.M., Santos Júnior L.S., Sousa K.S., da Silva Filho E.C. (2013). Dye anionic sorption in aqueous solution onto a cellulose surface chemically modified with aminoethanethiol. Chem. Eng. J..

[19] Ma J., Li F., Qian T., Liu H., Liu W., Zhao D. (2017). Natural organic matter resistant powder activated charcoal supported titanate nanotubes for adsorption of Pb(II). Chem. Eng. J..

[20] Ferreira F.J., Silva L.S., da Silva M.S., Osajima J.A., Meneguin A.B., Santagneli S.H., Barud H.S., Bezerra R.D., Silva-Filho E.C. (2019). Understanding kinetics and thermodynamics of the interactions between amitriptyline or eosin yellow and aminosilane-modified cellulose. Carbohydr. Polym..

[21] El-Hakam S., Ibrahim A.A., Elatwy L., El-Yazeed W.A., Salama R.S., El-Reash Y.A., Ahmed A.I. (2021). Greener route for the removal of toxic heavy metals and synthesis of 14-aryl-14H dibenzo[a,j] xanthene using a novel and efficient Ag-Mg bimetallic MOF as a recyclable heterogeneous nanocatalyst. J. Taiwan Inst. Chem. Eng..

[22] Bakry A.M., Alamier W.M., Salama R.S., El-Shall M.S., Awad F.S. (2022). Remediation of water containing phosphate using ceria nanoparticles decorated partially reduced graphene oxide (CeO2-PRGO) composite. Surf. Interfaces.

[23] Silva M.S., Silva L.S., Ferreira F.J., Bezerra R.D., Marques T.M., Meneguin A.B., Barud H.S., Osajima J.A., Silva Filho E.C. (2020). Study of interactions between organic contaminants and a new phosphated biopolymer derived from cellulose. Int. J. Biol. Macromol..

[24] Kukovecz Á., Kordás K., Kiss J., Kónya Z. (2016). Atomic scale characterization and surface chemistry of metal modified titanate nanotubes and nanowires. Surf. Sci. Rep..

[25] Li Z., Yang Y., Jáuregui-Haza U., Guo Z., Campos L.C. (2019). The impact of humic acid on metaldehyde adsorption onto powdered activated carbon in aqueous solution. RSC Adv..

[26] Vieira A.P., Santana S.A., Bezerra C.W., Silva H.A., de Melo J.C., da Silva Filho E.C., Airoldi C. (2010). Copper sorption from aqueous solutions and sugar cane spirits by chemically modified babassu coconut (Orbignya speciosa) mesocarp. Chem. Eng. J..

[27] Queiroga L.N., Pereira M.B., Silva L.S., Silva Filho E.C., Santos I.M., Fonseca M.G., Georgelin T., Jaber M. (2019). Microwave bentonite silylation for dye removal: Influence of the solvent. Appl. Clay Sci..

[28] De Castro Silva F., da Silva M.M.F., Lima L.C.B., Osajima J.A., da Silva Filho E.C. (2016). Integrating chloroethyl phosphate with biopolymer cellulose and assessing their potential for absorbing brilliant green dye. J. Environ. Chem. Eng..

[29] Lagergren S.K. (1898). About the theory of so-called adsorption of soluble substances. Sven. Vetenskapsakad. Handingarl.

[30] Ho Y., McKay G. (1999). Pseudo-second order model for sorption processes. Process Biochem..

[31] Elovich S.Y., Larinov O.G. (1962). Theory of adsorption from solutions of non electrolytes on solid (I) equation adsorption from solutions and the analysis of its simplest form,(II) verification of the equation of adsorption isotherm from solutions. Izv. Akad. Nauk. SSSR Otd. Khim. Nauk.

[32] Gusmão S.B., Ghosh A., Marques T.M., Gusmão G.O., Oliveira T.G., Cavalcante L.C.D., Vasconcelos T.L., Abreu G.J., Guerra Y., Peña-Garcia R. (2020). Structural and magnetic properties of titanate nano-heterostructures decorated with iron based nanoparticles. J. Phys. Chem. Solids.

[33] Sales D.A., Marques T.M., Ghosh A., Gusmão S.B., Vasconcelos T.L., Luz-Lima C., Ferreira O.P., Hollanda L.M., Lima I.S., Silva-Filho E.C. (2020). Synthesis of silver-cerium titanate nanotubes and their surface properties and antibacterial applications. Mater. Sci. Eng. C.

[34] Gusmão S.B., Ghosh A., Marques T.M., Vieira L.H.S., Ferreira O.P., Silva-Filho E.C., Lobo A.O., Osajima J.A., Viana B.C. (2019). Titanate-based one-dimensional nano-heterostructure: Study of hydrothermal reaction parameters for improved photocatalytic application. Solid State Sci..

[35] Gusmão S.B.S., Ghosh A., Marques T.M.F., Ferreira O.P., Lobo A.O., Osajima J.A.O., Luz-Lima C., Sousa R.R.M., Matos J.M.E., Viana B.C. (2019). One-Pot Synthesis of Titanate Nanotubes Decorated with Anatase Nanoparticles Using a Microwave-Assisted Hydrothermal Reaction. J. Nanomater..

[36] Chen Q., Du G., Zhang S., Peng L.M. (2002). The structure of trititanate nanotubes. Acta Crystallogr. Sect. B Struct. Sci..

[37] Viana B.C., Ferreira O.P., Souza Filho A.G., Hidalgo A.A., Mendes Filho J., Alves O.L. (2011). Highlighting the mechanisms of the titanate nanotubes to titanate nanoribbons transformation. J. Nanopart. Res..

[38] Pontón P.I., D’Almeida J.R., Marinkovic B.A., Savić S.M., Mancic L., Rey N.A., Morgado E., Rizzo F.C. (2014). The effects of the chemical composition of titanate nanotubes and solvent type on 3-aminopropyltriethoxysilane grafting efficiency. Appl. Surf. Sci..

[39] Plodinec M., Gajović A., Iveković D., Tomašić N., Zimmermann B., Macan J., Haramina T., Su D.S., Willinger M. (2014). Study of thermal stability of (3-aminopropyl)trimethoxy silane-grafted titanate nanotubes for application as nanofillers in polymers. Nanotechnology.

[40] Brnardić I., Huskić M., Umek P., Fina A., Grgurić T.H. (2013). Synthesis of silane functionalized sodium titanate nanotubes and their influence on thermal and mechanical properties of epoxy nanocomposite. Phys. Status Solidi.

[41] Maleki A., Hamesadeghi U., Daraei H., Hayati B., Najafi F., McKay G., Rezaee R. (2017). Amine functionalized multi-walled carbon nanotubes: Single and binary systems for high capacity dye removal. Chem. Eng. J..

[42] Silva L.S., Lima L.C.B., Ferreira F.J.L., Silva M.S., Osajima J.A., Bezerra R.D.S., Filho E.C.S. (2015). Sorption of the anionic reactive red RB dye in cellulose: Assessment of kinetic, thermodynamic, and equilibrium data. Open Chem..

[43] Silva L.S., Ferreira F.J., Silva M.S., Citó A.M., Meneguin A.B., Sábio R.M., Barud H.S., Bezerra R.D., Osajima J.A., Silva Filho E.C. (2018). Potential of amino-functionalized cellulose as an alternative sorbent intended to remove anionic dyes from aqueous solutions. Int. J. Biol. Macromol..

[44] Monsef Khoshhesab Z., Modaresnia N. (2019). Adsorption of Acid Black 210 and Remazol Brilliant Blue R onto magnetite nanoparticles. Inorg. Nano-Met. Chem..

[45] Monsef Khoshhesab Z., Souhani S. (2018). Adsorptive removal of reactive dyes from aqueous solutions using zinc oxide nanoparticles. J. Chin. Chem. Soc..

[46] Silva T.L., Ronix A., Pezoti O., Souza L.S., Leandro P.K., Bedin K.C., Beltrame K.K., Cazetta A.L., Almeida V.C. (2016). Mesoporous activated carbon from industrial laundry sewage sludge: Adsorption studies of reactive dye Remazol Brilliant Blue R. Chem. Eng. J..

[47] Jawad A.H., Abdulhameed A.S., Kashi E., Yaseen Z.M., ALOthman Z.A., Khan M.R. (2022). Cross-Linked Chitosan-Glyoxal/Kaolin Clay Composite: Parametric Optimization for Color Removal and COD Reduction of Remazol Brilliant Blue R Dye. J. Polym. Environ..

[48] Reghioua A., Barkat D., Jawad A.H., Abdulhameed A.S., Rangabhashiyam S., Khan M.R., ALOthman Z.A. (2021). Magnetic Chitosan-Glutaraldehyde/Zinc Oxide/Fe3O4 Nanocomposite: Optimization and Adsorptive Mechanism of Remazol Brilliant Blue R Dye Removal. J. Polym. Environ..

[49] Bouzikri S., Ouasfi N., Benzidia N., Salhi A., Bakkas S., Khamliche L. (2020). Marine alga “Bifurcaria bifurcata”: Biosorption of Reactive Blue 19 and methylene blue from aqueous solutions. Environ. Sci. Pollut. Res..

[50] Guerritore M., Castaldo R., Silvestri B., Avolio R., Cocca M., Errico M.E., Avella M., Gentile G., Ambrogi V. (2020). Hyper-Crosslinked Polymer Nanocomposites Containing Mesoporous Silica Nanoparticles with Enhanced Adsorption Towards Polar Dyes. Polymers.

[51] Mate C.J., Mishra S. (2020). Synthesis of borax cross-linked Jhingan gum hydrogel for remediation of Remazol Brilliant Blue R (RBBR) dye from water: Adsorption isotherm, kinetic, thermodynamic and biodegradation studies. Int. J. Biol. Macromol..

[52] Chinoune K., Bentaleb K., Bouberka Z., Nadim A., Maschke U. (2016). Adsorption of reactive dyes from aqueous solution by dirty bentonite. Appl. Clay Sci..

[53] Shanehsaz M., Seidi S., Ghorbani Y., Shoja S.M.R., Rouhani S. (2015). Polypyrrole-coated magnetic nanoparticles as an efficient adsorbent for RB19 synthetic textile dye: Removal and kinetic study. Spectrochim. Acta Part A Mol. Biomol. Spectrosc..

